# Traditional Food Use and Ethnomedicinal Contexts of Turnip (*Brassica rapa* L.): A Scoping Review of Composition, Food Processing, and Health-Related Evidence

**DOI:** 10.3390/foods15203648

**Published:** 2026-10-14

**Authors:** Kanxin Ye, Hua Zhang, Huiqiang Hu, Yaohui Ye, Lihua Chen, Yun Xiong

**Affiliations:** 1 Department of Development and Evaluation of Dietary Functional Products, Key Laboratory of Modern Preparation of Chinese Medicine, Ministry of Education, Jiangxi University of Chinese Medicine, Nanchang 330004, China; yekanxin@jxutcm.edu.cn (K.Y.); 20191002@jxutcm.edu.cn (H.Z.); huhuiqiang@jxutcm.edu.cn (H.H.); 2 Nanchang Medical College, Nanchang 330038, China; yeyaohui791@ncmc.edu.cn

**Keywords:** *Brassica rapa*, turnip, Qiamagu, ethnomedicine, phytochemicals, food processing, bioactivity, scoping review

## Abstract

Turnip (*Brassica rapa* L.) is widely cultivated for food and has documented ethnomedicinal uses. This scoping review mapped the international literature on turnip, with detailed synthesis focused on turnip materials associated with selected regional food and ethnomedicinal traditions in China. Searches covered seven bibliographic databases. The bibliographic map comprised 771 records, of which 397 were selected for detailed synthesis. Regional uses varied in plant part and preparation method. The clearest evidence concerned composition and processing: constituent profiles differed by provenance, plant part, and analytical method, whereas changes in composition and product quality depended on the product and process. Animal and cell studies reported effects of specific extracts, fractions, and multicomponent formulations on hypoxia tolerance, endurance, tissue injury, inflammation, redox regulation, metabolism, and the gut microbiota, but mechanistic findings did not converge across preparations and models. The few human studies addressed mainly hypoxia or exercise and supportive care during chemotherapy; differences in design, preparations, and outcomes precluded reproducible conclusions about any defined turnip food or ethnomedicinal preparation. Overall, this review clarifies the current evidence on turnip as a food resource, documents its ethnomedicinal contexts, and identifies priorities for future research and product development.

## 1. Introduction

Turnip (*Brassica rapa* L.) is a widely cultivated food crop in the family Brassicaceae. Sources from Europe, Western Asia, the Caucasus, and trans-Himalayan Asia document consumption of the fleshy root (the swollen hypocotyl–root storage organ) and, in some communities, the leaves, whether raw, cooked, fermented, pickled, or dried [1,2,3,4]. An ethnobotanical study from the Aosta Valley, Italy, also records sugar-treated root slices as a household cough preparation [5].

Within this global context, the literature from China documents a broad range of regional names, foods, and ethnomedicinal uses. In Xinjiang, Qiamagu or qamgur features in local diets and Uyghur dietary therapy [6,7,8]. In Liangshan, Sichuan, yuangen is used in a traditional Yi fermented food [9]. In the Xizang Autonomous Region, “Tibetan turnip” or Manjing occurs in Tibetan medicinal preparations [10,11]. A regional food standard also applies to turnip [12]. In northwestern Yunnan, Naxi communities use Manjing, Wu Jing, or A Ke as food and in traditional medicine [13,14,15].

Research on turnip encompasses foods, crude extracts, fractions, and multicomponent formulations. The fleshy root is the most frequently studied material, although root peel, aerial tissues, seeds, and seed oil have also been examined [8,16,17,18]. Food studies address composition, processing, and product quality, whereas most health-related experiments test extracts or fractions in animals or cells. The latter findings concern the tested preparations rather than the consumption of turnip foods.

Several reviews have addressed aspects of turnip research [19,20,21,22]. Nevertheless, evidence on its traditional food and ethnomedicinal uses and its value as a food resource remains scattered across regional and disciplinary studies. A focused synthesis is therefore needed to clarify turnip’s food value and support subsequent research and product development.

This scoping review combines an international bibliographic map with a detailed synthesis of turnip materials associated with selected regional food and ethnomedicinal traditions in China. This regional focus was informed by the concentration of China-associated research and its links to documented food and ethnomedicinal uses. The review examines composition and processing in relation to food quality and interprets health-related findings according to the materials and study designs used, with the aim of advancing turnip research and food-product development.

## 2. Materials and Methods

### 2.1. Review Design and Eligibility Criteria

This scoping review incorporated an evidence-mapping component. The design drew on guidance from the Joanna Briggs Institute (JBI) [23]. Reporting followed the Preferred Reporting Items for Systematic Reviews and Meta-Analyses extension for Scoping Reviews (PRISMA-ScR) and the PRISMA extension for literature searches (PRISMA-S) [24,25]. The protocol was documented during review conduct and deposited in a private Open Science Framework (OSF) project on 5 July 2026, before final eligibility verification and synthesis. Targeted supplementary checks followed in August 2026.

Eligibility criteria were structured using the Population–Concept–Context framework. The Population included *Brassica rapa* materials identifiable as turnip and preparations derived from them, including the fleshy root and other organs explicitly obtained from turnip plants. The Concept encompassed traditional uses, composition and nutrition, extraction, food processing and food products, biological activity, and health-related research. The Context covered food, nutrition, functional-food research, and ethnomedicinal uses of edible plants. Other *B. rapa* crop groups and records using only a broad *B. rapa* designation were excluded unless turnip-specific results could be identified separately. When regional terminology, plant part, local use, and provenance collectively indicated turnip but botanical verification remained incomplete, the source terminology was retained and the material was classified as having uncertain identity.

All records meeting the species and topic criteria were included in the global bibliographic map. Detailed synthesis focused on selected Chinese regional turnip traditions, centered on Xinjiang qiamagu and plateau turnip (yuangen), and included related regional food preparations, ethnomedicinal materials, and comparative studies. Selection within this regional focus was based on material provenance, regional names, or documented uses, rather than publication language or author affiliation alone. Other eligible records from China and elsewhere were retained for contextual mapping; they contributed to the regional-use synthesis only when the species, plant part, preparation, and geographic practice could be verified. Eligible sources included original studies, reviews, theses, conference papers, and surveys of plant resources. Detailed results were extracted from complete original reports with sufficient methodological and outcome information. Conference abstracts without sufficient methodological and outcome information were retained for bibliographic description rather than independent outcome synthesis. Studies concerned solely with agronomy, breeding, genetics, plant pathology, environmental toxicology, phytoremediation, or plant viruses were excluded if they provided no relevant information on food, composition, nutrition, or health. Official documents, standards, patents, and registries of scientific and technological achievements were used only to clarify nomenclature, product status, or institutional context. Findings from multicomponent studies were attributed to the complete formulation unless the study design supported attribution to a specific component.

### 2.2. Information Sources and Search Strategy

Seven bibliographic databases were searched between 17 June and 5 July 2026, from database inception through the final search date: Web of Science Core Collection, PubMed, Scopus, China National Knowledge Infrastructure, Wanfang Data, the VIP Chinese Science and Technology Journal Database (VIP/CQVIP), and the Chinese Biomedical Literature Database (CBM) via SinoMed. Database-specific strategies combined scientific, common, and regional names with terms related to composition, nutrition, processing, products, biological activity, and health; no language or geographic limits were applied. Google Scholar was used as a supplementary search source. Reference lists were screened, and targeted searches were conducted for ethnobotanical records and by author, journal, and publisher platform to identify potentially missed records and verify source details. Complete search strategies, dates, yields, limits, and search logs are provided in the PRISMA-S supplement.

### 2.3. Study Selection and Data Charting

**Figure 1:**
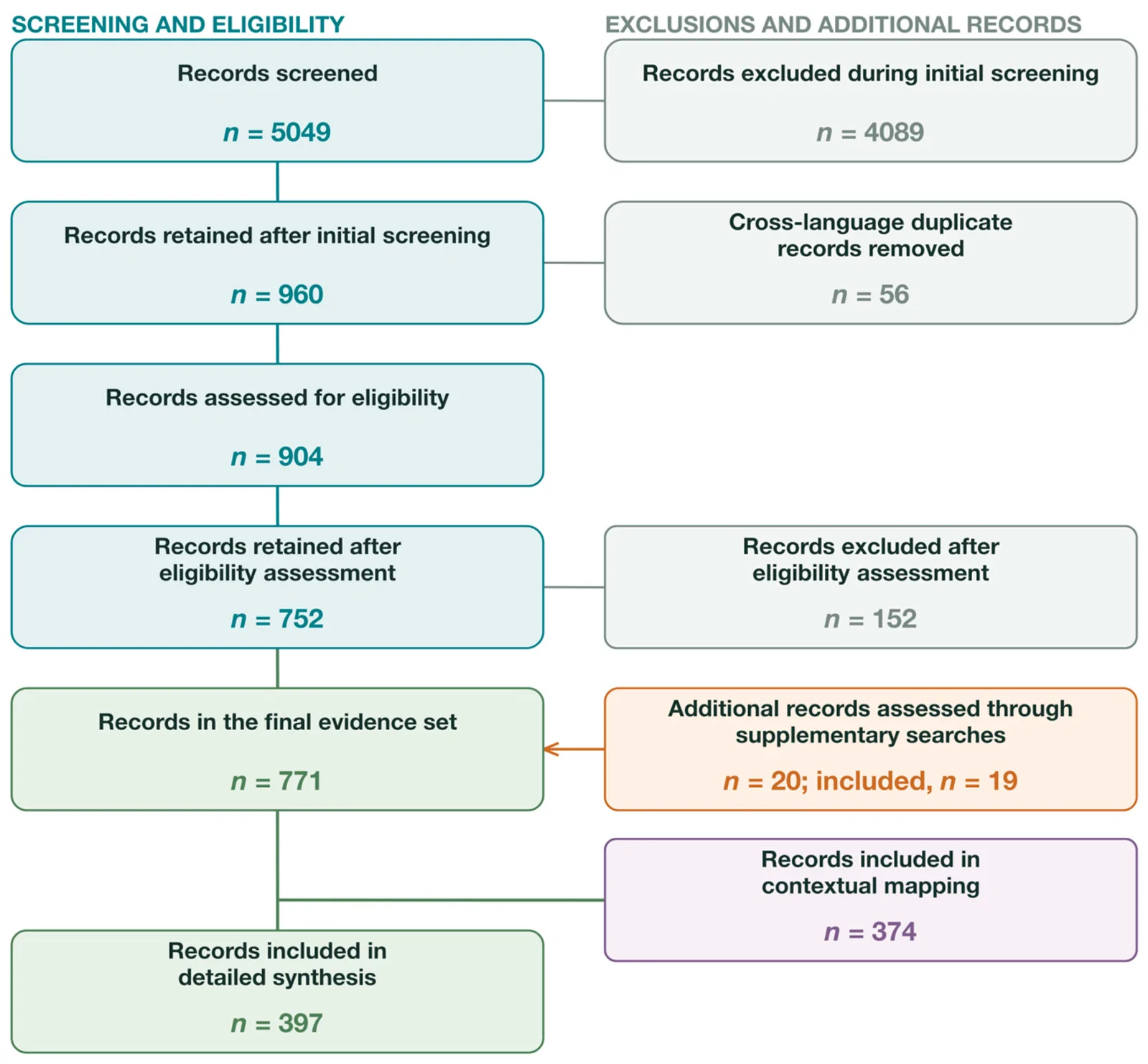
Identification, screening, eligibility assessment, and final use of 771 bibliographic records. The flow diagram shows record consolidation, eligibility screening, supplementary searches, and allocation to detailed synthesis (*n* = 397) or contextual mapping (*n* = 374). Counts refer to bibliographic records.

After initial relevance screening within each source, records were merged and obvious duplicates removed, leaving 5049 records for title and abstract screening. Screening excluded records that did not concern turnip or relevant food, compositional, nutritional, or health topics. Remaining cross-database and cross-language duplicates were reconciled before eligibility assessment. Two reviewers independently screened records and assessed eligibility according to the stated criteria, resolving disagreements through discussion. Full texts or complete source documents were obtained for all 404 records initially selected for detailed assessment and used to verify eligibility and target-material identity. Records used for contextual mapping were assessed from titles, abstracts, keywords, and bibliographic metadata. The same criteria applied to supplementary checks. Figure 1 and the PRISMA-S supplement provide the screening counts.

A pilot-tested form was used to chart the material and preparation, study design, comparators, outcomes, mechanisms, and major limitations for records in the detailed synthesis; all entries were checked by a second reviewer. Each record was assigned one primary study type and could be classified under multiple topics. Contextual-mapping records were not used in detailed analyses of experimental outcomes. Information on regional uses could be drawn from either evidence layer only when the target species, plant part, preparation, and geographic practice were verifiable.

Chinese full texts were read and data extracted directly by the authors, with Eudic (version 26.9.1) and Youdao Translate (version 11.3.20) used as translation aids. English wording was checked against the original source. Published English titles and abstracts were used where available; otherwise, bibliographic information was translated and checked against the original-language entries retained in Table S1.

### 2.4. Data Synthesis

Publication characteristics and the distributions of study types and topics were summarized using frequencies and proportions. Findings on regional uses, composition, processing, and health-related outcomes were synthesized narratively according to material provenance, plant part, preparation, study design, and outcome [23]. Quantitative compositional data were compared only when the materials and analytical conditions were compatible. Documented traditional foods and preparations were synthesized separately from modern experimental preparations. Reviews were used for contextual background and to identify primary sources; when a review described an eligible primary experiment, only the corresponding original report was counted.

Nonhuman bioactivity data were synthesized at the level of individual experiments. Experiments were treated separately when they differed in test material, model, exposure, or comparator. Multiple outcomes from the same experiment were extracted separately. Linked conference, thesis, and journal reports were grouped as one experiment when they described the same work, with data extracted from the most complete report. Highly similar experiments were not counted independently when their independence could not be established. Mechanistic evidence was classified as direct only when a candidate mediator was experimentally manipulated and the functional outcome was reassessed in the same experiment. Pathway-level, omics, and computational findings without such manipulation were treated as hypothesis-generating. Experiment-level summaries and the criteria used to interpret mechanistic evidence are provided in Table S4.

## 3. Results and Discussion

### 3.1. Study Selection and Evidence Characteristics

#### 3.1.1. Search Results and Included Records

After initial relevance screening within each source, records were merged and obvious duplicates removed, leaving 5049 records for title and abstract screening. Screening excluded 4089 records that did not meet the species or topic criteria, leaving 960. Reconciliation of 42 cross-language duplicate groups removed a further 56 records, leaving 904 for eligibility and target-material verification; 152 were not retained. Supplementary checks assessed 20 additional records and retained 19, yielding a final bibliographic map of 771 records (Figure 1).

All 771 records were included in bibliographic mapping of terminology, publication patterns, and the geographic distribution of the literature. Among them, 397 records underwent detailed data charting within the regional-material scope described in Section 2.1 and supported the syntheses of composition, processing, biological activity, and human evidence. The other 374 records were used for contextual mapping; those with verifiable species identity, plant part, preparation, and regional practice also informed the regional-use synthesis (Table S1; Figure 1).

#### 3.1.2. Publication Trends and First-Author Affiliations

**Figure 2:**
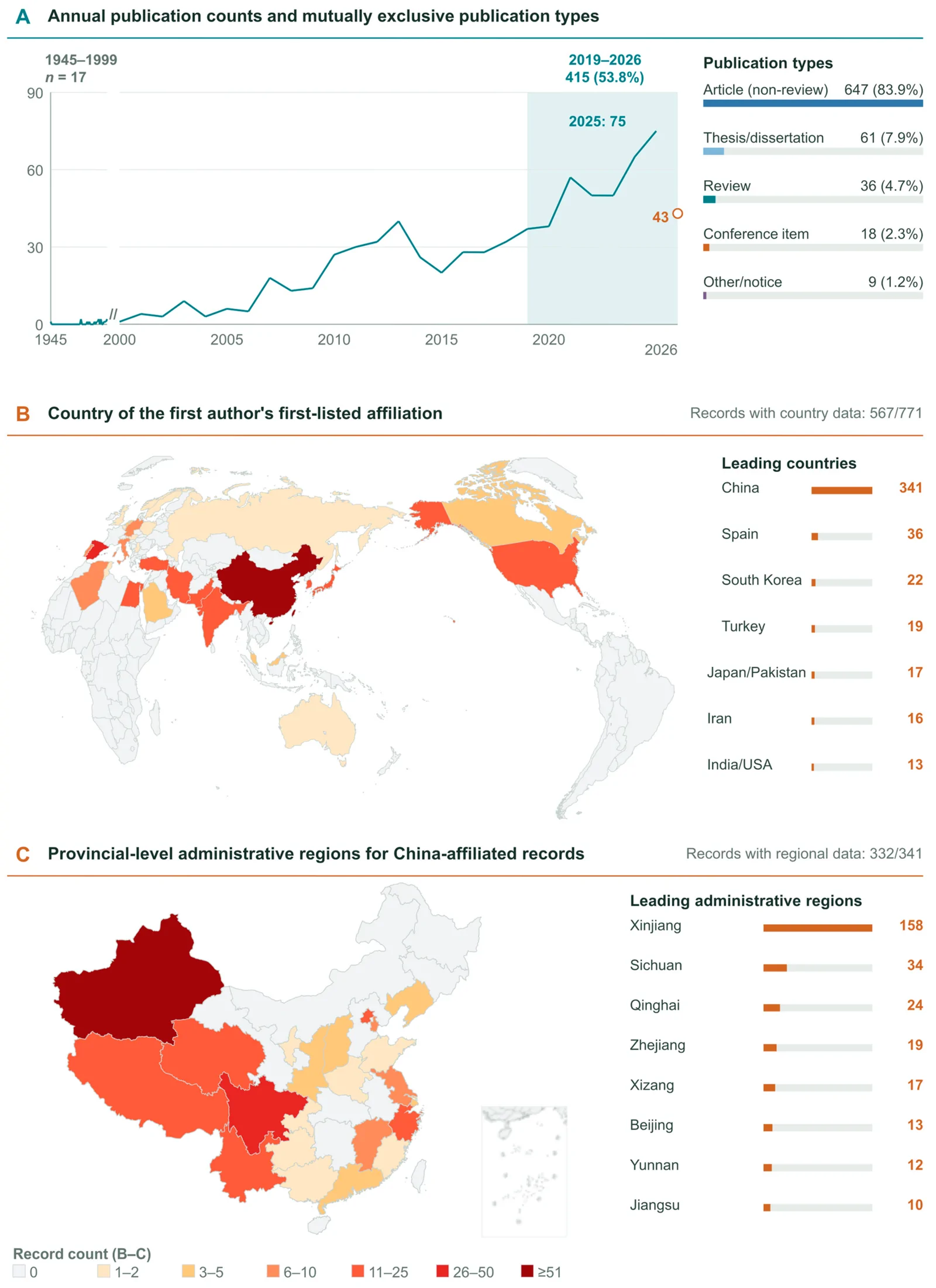
Publication trends, publication types, and geographic distribution of first-author affiliations among 771 records. (**A**) Annual record counts and publication types. Bar colors distinguish publication types; the orange open circle and label mark the 2026 count through the final search date. (**B**) Country of the first-listed affiliation of the first author for 567 records with identifiable affiliation-country data. (**C**) Distribution across provincial-level administrative regions of China for 332 of the 341 China-affiliated records. Base maps for panels (**B**,**C**) were adapted from GS(2021)5450 and GS(2022)4301, respectively, provided by the Standard Map Service System of the Ministry of Natural Resources of China. Panels (**B**,**C**) show author affiliations rather than the origins of materials, studies, or participants. Publication-type counts include contextual sources and do not indicate numbers of independent experiments.

**Figure 3:**
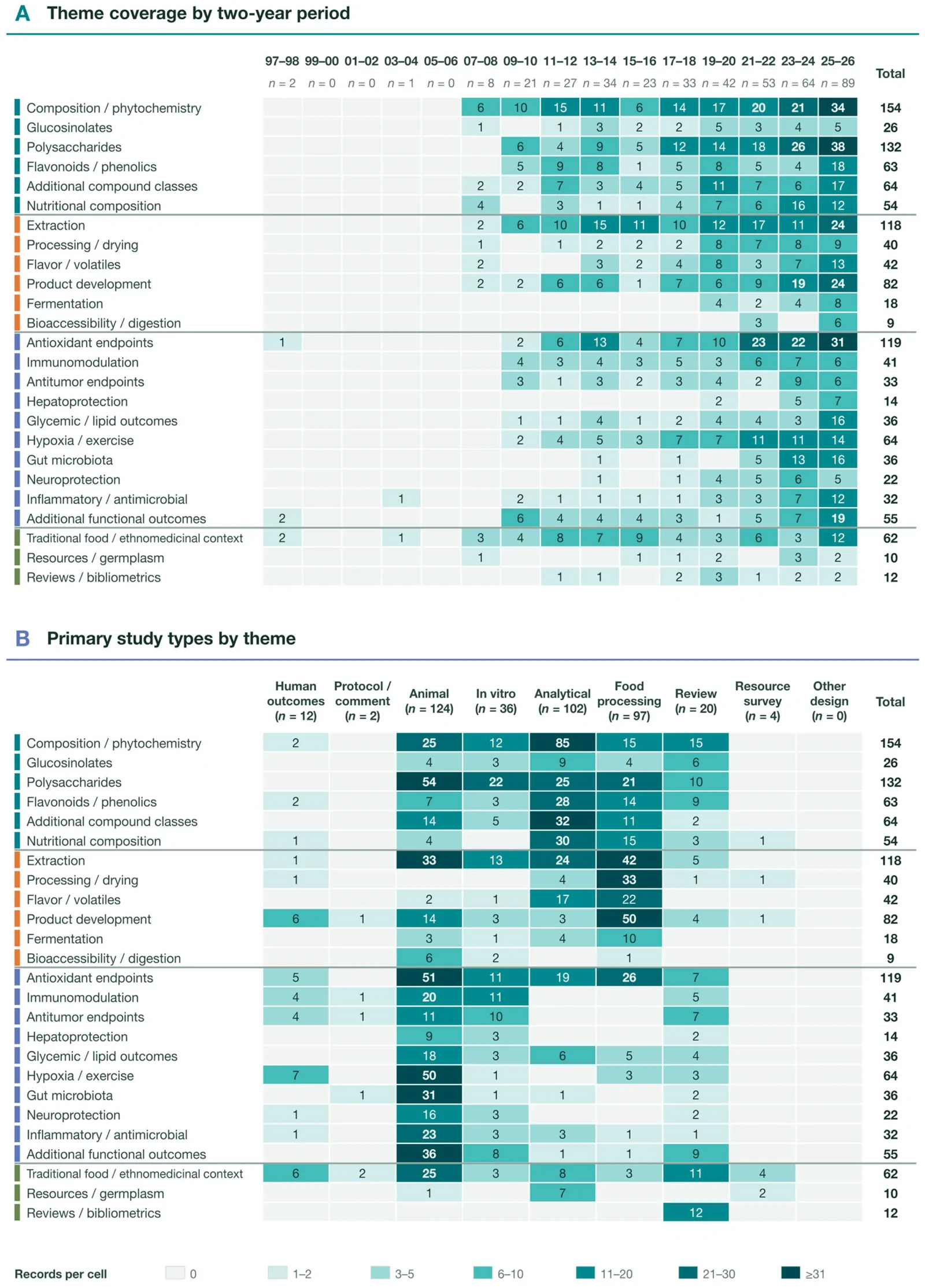
Thematic coverage and primary study types among 397 records included in detailed synthesis. (**A**) Non-mutually exclusive themes across 15 consecutive two-year periods. Data for 2026 are incomplete; the main database searches ended on 5 July 2026. (**B**) Mutually exclusive primary study types within each theme. Each record could contribute to multiple themes but had one primary study type; cell color indicates the number of records.

The final bibliographic map comprised 647 non-review journal articles, 36 reviews, 61 theses or dissertations, 18 conference papers or abstracts, and 9 other sources or notices (Figure 2; Table S1). Records published from 2019 onward totaled 415, representing 53.8% of the full record set; 248 were included in the detailed synthesis and accounted for 62.5% of the 397 detailed records. Publications from 2019 onward therefore accounted for more than half of both the full record set and the detailed-synthesis set. Because the principal database searches ended in July 2026, the 2026 counts represent an incomplete publication year (Figure 2A and Figure 3A; Table S1).

The country of the first author’s first-listed institutional affiliation was identifiable for 567 records; for 341 records, that affiliation was in China, while the remainder represented multiple countries. Among the 332 records with a China-based affiliation and province-level information, Xinjiang was most frequent, followed by Sichuan, Qinghai, Zhejiang, the Xizang Autonomous Region, Beijing, and Yunnan. These counts reflect the locations of research institutions rather than the geographic origins of study materials or participants. Figure 2B,C presents the affiliation data; material provenance, study location, and participant origin were analyzed separately.

#### 3.1.3. Study Types, Topics, and Changes over Time

Among the 397 records in the detailed synthesis, the mutually exclusive study-type classification identified 124 animal studies, 102 analytical studies, and 97 processing studies; together, these categories accounted for 323 records (81.4%). A further 36 were in vitro studies. Twelve records reported human outcomes. Two additional records were protocols or commentaries without study results. The remaining 24 records comprised 20 reviews and four surveys of plant resources (Figure 3B). The detailed evidence base was therefore dominated by analytical, processing, animal, and in vitro research, whereas human studies represented only a small proportion.

Topic counts describe the distribution of research, not the strength or independent replication of its findings. Records could address multiple topics, so these counts were not mutually exclusive. Composition and phytochemistry (154 records), polysaccharides (132), antioxidant assessment (119), extraction (118), and product development (82) were the most commonly assigned topics. Traditional food use, ethnomedicinal use, or related regional context was identified in 62 records, while 10 addressed resource or germplasm topics. Growth after 2019 occurred mainly in composition, extraction, processing, and experimental-model research. Fermentation (18 records) and bioaccessibility or digestion (9) were represented mainly in later publications and remained uncommon, while human studies continued to form only a small component of the evidence base (Figure 3A,B).

### 3.2. Material Identity, Provenance, and Preparation

#### 3.2.1. Names, Taxonomy, and Material Sources

Studies reported materials under names including Qiamagu, turnip, and yuangen. Plants of the World Online (POWO) accepts *Brassica rapa* L. at the species level. The turnip form is treated as *B. rapa* var. *rapa* in Flora of China, whereas the World Flora Online (WFO) taxonomic backbone lists it as *B. rapa* subsp. *rapa* and places *B. rapa* var. *rapa* in synonymy under that subspecies [26,27,28]. Throughout this review, turnip denotes the turnip form of *B. rapa*; regional names are retained when needed to specify material identity or context of use. Current place names are used for geographic provenance, whereas source-specific material names such as “Tibetan turnip” and ethnomedicinal terms are retained as published.

Historical names were checked against current taxonomic relationships. A composite-beverage study from Keping, Xinjiang, China, identified its material as *B. campestris*, which POWO treats as a synonym of *B. rapa* [29,30]. Records explicitly using *B. napobrassica* were excluded because POWO places that name in synonymy under *B. napus* [31]. Turnip identity was assessed from voucher specimens, identification by a named botanist, documented seed or cultivar sources, or adequate morphological information.

Provenance reporting ranged from detailed voucher documentation to market purchase without a reported production history. A Keping study named the botanist who identified the material but did not report a voucher specimen, cultivar, or independent collection batches [32]. Root material purchased in Lhasa, the Xizang Autonomous Region, China, and described as “Tibetan turnip” was authenticated by a named botanist and deposited as voucher B161019 [33]. *Brassica rapa* var. *rapa* collected in Lanping, Yunnan, China, was documented as voucher KTRG-B-43 [34]. By contrast, material purchased at a market in Shihezi, Xinjiang, China, had a documented point of acquisition but no reported production origin, cultivar, or voucher specimen [35]. The records therefore differed substantially in the traceability of botanical identity and provenance.

Material origin, experimental site, and author affiliation are distinct geographic attributes. One study compared root samples supplied from nine counties in Qamdo, the Xizang Autonomous Region, China, but did not report independent biological replication within each county [36]. Another study grew 50 accessions from different source regions at a single experimental site in Qinghai, China [37]. The former compared batches collected from different locations, whereas the latter compared accessions under common cultivation. These designs address different sources of variation and do not independently separate accession, environment, and sampling-batch effects.

#### 3.2.2. Plant Parts and Preparation Levels

The literature extended beyond the fleshy root to include peels from green- and purple-skinned turnips, distillates prepared separately from roots and aerial stems and leaves, and oils obtained from turnip seeds [16,17,18]. The same plant part could also undergo different treatments, including five drying procedures used to produce powders, alternative dehydration routes before natural salt fermentation, and raw, boiled, steamed, or microwaved preparation of material from the same batch [35,38,39]. The plant part and processing state therefore jointly defined the material examined.

Preparation levels ranged from intact tissue or powder to crude extracts, enriched fractions, enzymatically generated mixtures, purified macromolecules, and isolated compounds. A preparation described as “Qiamagu saponins” was a crude 70% ethanol extract in which no individual saponin was confirmed [40]. Other studies tested an enzymatically generated isothiocyanate mixture, a purified acidic polysaccharide fraction, or compounds isolated from root material documented by a voucher specimen [41,42,43]. Extracted fractions and isolated compounds differ from foods in composition and exposure; analytical standards served only as calibration or conversion references [44].

**Figure 4:**
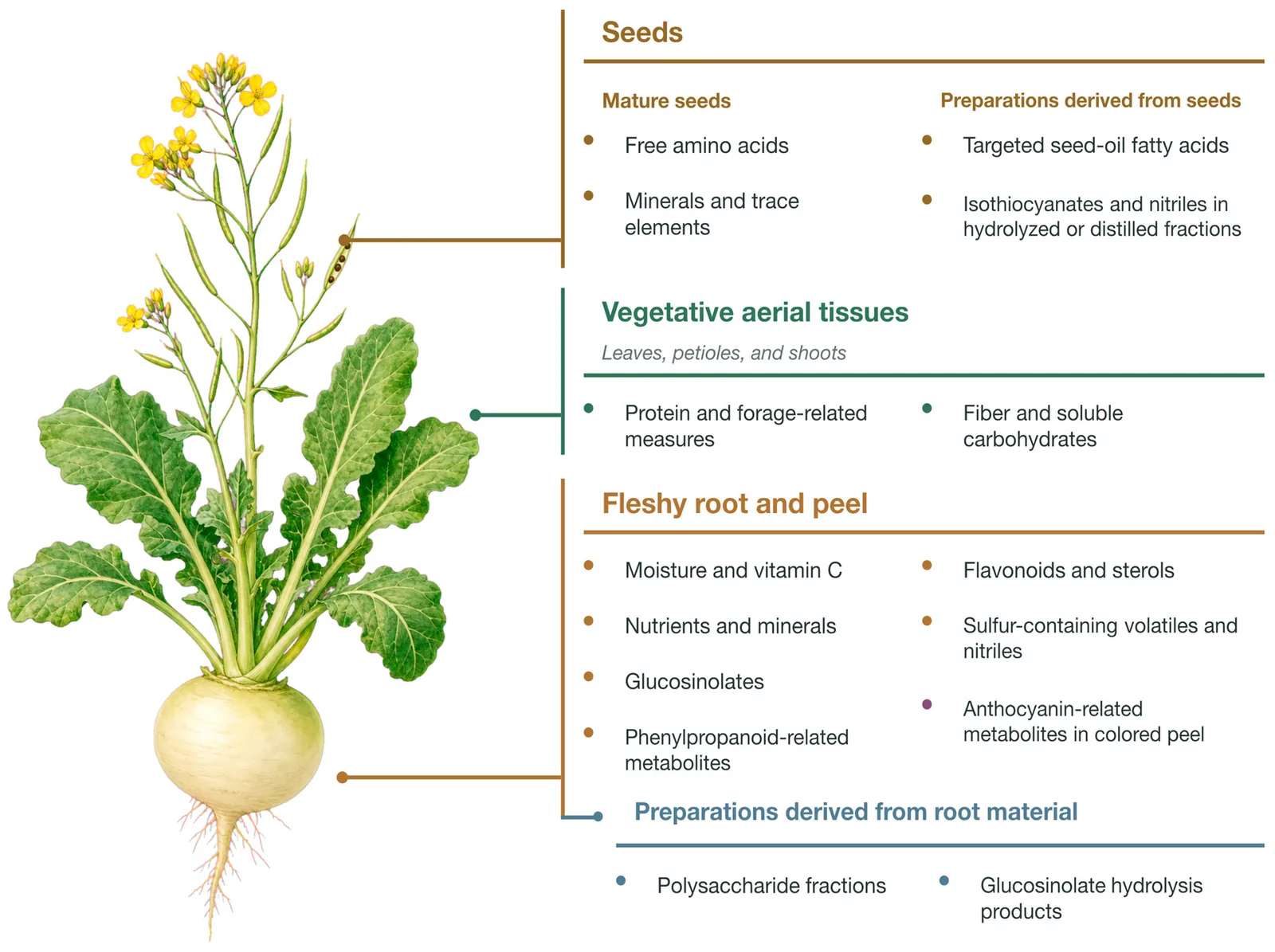
Turnip plant parts and preparation levels represented in compositional studies. Seeds, aerial tissues, fleshy roots and peels, and seed- or root-derived preparations are shown separately to distinguish the constituents reported for each material type. Selected illustrative elements were generated using OpenAI image-generation tools.

Multicomponent products were interpreted as complete formulations. One beverage combined Qiamagu root pulp with apricot, jujube, grape, and other ingredients. In another study, yuangen appeared only in an initial screening formulation and was absent from the final formulation used for the cellular assays [29,45]. Attribution to turnip requires a comparator that isolates its contribution from the other ingredients. Figure 4 summarizes the principal plant parts and preparation levels represented in compositional studies.

#### 3.2.3. Regional Food and Ethnomedicinal Contexts

Regional-use records documented turnip foods and reported ethnomedicinal practices across Asia, Europe, North Africa, and the Caucasus. These accounts describe use rather than demonstrate therapeutic efficacy. Plant parts and preparation methods varied by location, but fermented or pickled foods, raw or cooked roots, edible leaves, and household or ethnomedicinal preparations formed recurrent patterns. Among the 397 records in detailed synthesis, 62 addressed traditional food use, ethnomedicinal use, or related regional contexts. Verified contextual-mapping records also contributed to this regional synthesis (Figure 3A; Table S1).

Fermentation, pickling, and storage were recurring food practices, although the materials and processes were region specific. A study from Liangshan Yi Autonomous Prefecture, Sichuan, China, documented a traditional Yi fermented yuangen vegetable, distinguished the handling of stems and leaves from that of the root, and evaluated leaves and leaf veins in the finished product [9]. Related studies examined salting, spontaneous fermentation, air-drying or salt dehydration, and fermentation or pickling over periods of 1–28 days [39,46]. In Eastern Tyrol, Austria, chopped roots were fermented without salt for three to five weeks [1]. Sources from Ladakh, India, documented stored roots, sun-dried leaves, and fermented pickles [2], while sources from Türkiye described boiled or pickled roots and dishes or preserved foods prepared from roots and leaves [3,47]. Together, these records show how plant part, salt use, fermentation time, and subsequent handling define distinct regional foods.

Raw, cooked, dried, and stored foods extended beyond fermented preparations. Two linked surveys among Naxi communities in Lijiang, northwestern Yunnan, China, documented raw or boiled Manjing root together with the drying, storage, or pickling of fleshy roots and leaves [13,14]. A survey in Talagang, Pakistan, supported by deposited voucher specimens, recorded roots and leaves cooked with onion, garlic, tomato, and coriander as a meal [48]. Among Monpa communities in Tawang, Arunachal Pradesh, India, the fleshy root was eaten raw or cooked [49]. A source from Georgia in the Caucasus identified the root as food without further preparation detail [4], and a survey in northwestern Algeria documented edible leaves [50]. These examples broaden the regional food record from the fleshy root to leaves and from fresh consumption to storage and preservation.

**Figure 5:**
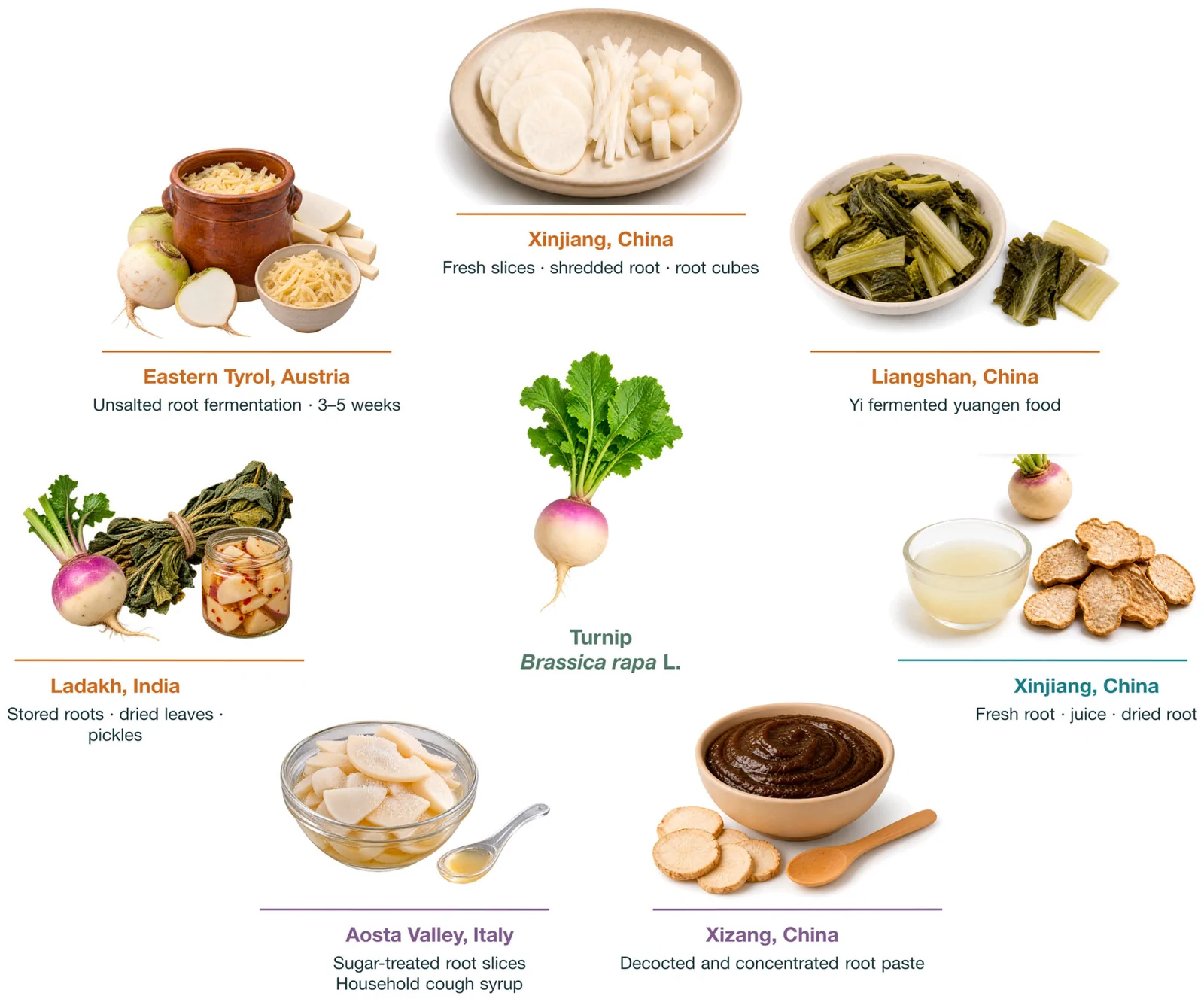
Source-documented regional turnip foods and preparations used in dietary therapy, ethnomedicine, or household care: unsalted fermented root from Eastern Tyrol, Austria [1]; stored roots, dried leaves, and pickles from Ladakh, India [2]; sugar-treated root slices used as a household cough syrup in the Aosta Valley, Italy [5]; fresh-root foods and dietary-therapy preparations from Xinjiang, China [8,21]; Yi fermented yuangen food from Liangshan Yi Autonomous Prefecture, Sichuan, China [9]; and turnip paste used in Tibetan medicine in the Xizang Autonomous Region, China [10,11]. Selected illustrative elements were generated using OpenAI image-generation tools.

Ethnomedicinal and household uses were likewise tied to identifiable plant parts and preparation methods. A study of Naxi Dongba sutras combined textual analysis with interviews of 104 folk healers and linked the Chinese name Wu Jing and the Naxi name A Ke to a *B. rapa* root documented by a voucher specimen; the dried root was taken orally for bladder symptoms [15]. Xinjiang sources documented Qiamagu or qamgur in local diets and dietary therapy within Uyghur medicine [6,7,8,21]. Sources from the Xizang Autonomous Region described cleaned and dried fleshy roots or root slices that were decocted and concentrated into Tibetan medicinal turnip paste [10,11]. A survey in Kerman, Iran, recorded oral use of the edible root for respiratory complaints and the common cold [51], whereas the Algerian survey documented fresh, dried, heated, infused, decocted, powdered, and topical uses of leaves [50]. In the Aosta Valley, Italy, sugar-treated root slices were prepared as a household cough syrup [5]. Figure 5 summarizes these region-specific foods and preparations.

Policies, geographical-indication documents, and local standards further documented regional naming and product definitions [12,52,53,54]. In China, turnip is not included in the current official list of substances traditionally used as both foods and Chinese medicinal materials [55,56,57,58,59,60]. These documents distinguish regional naming and product standards from inclusion in China’s national category of substances traditionally used as both food and Chinese medicinal materials.

### 3.3. Phytochemical and Nutritional Composition

**Figure 6:**
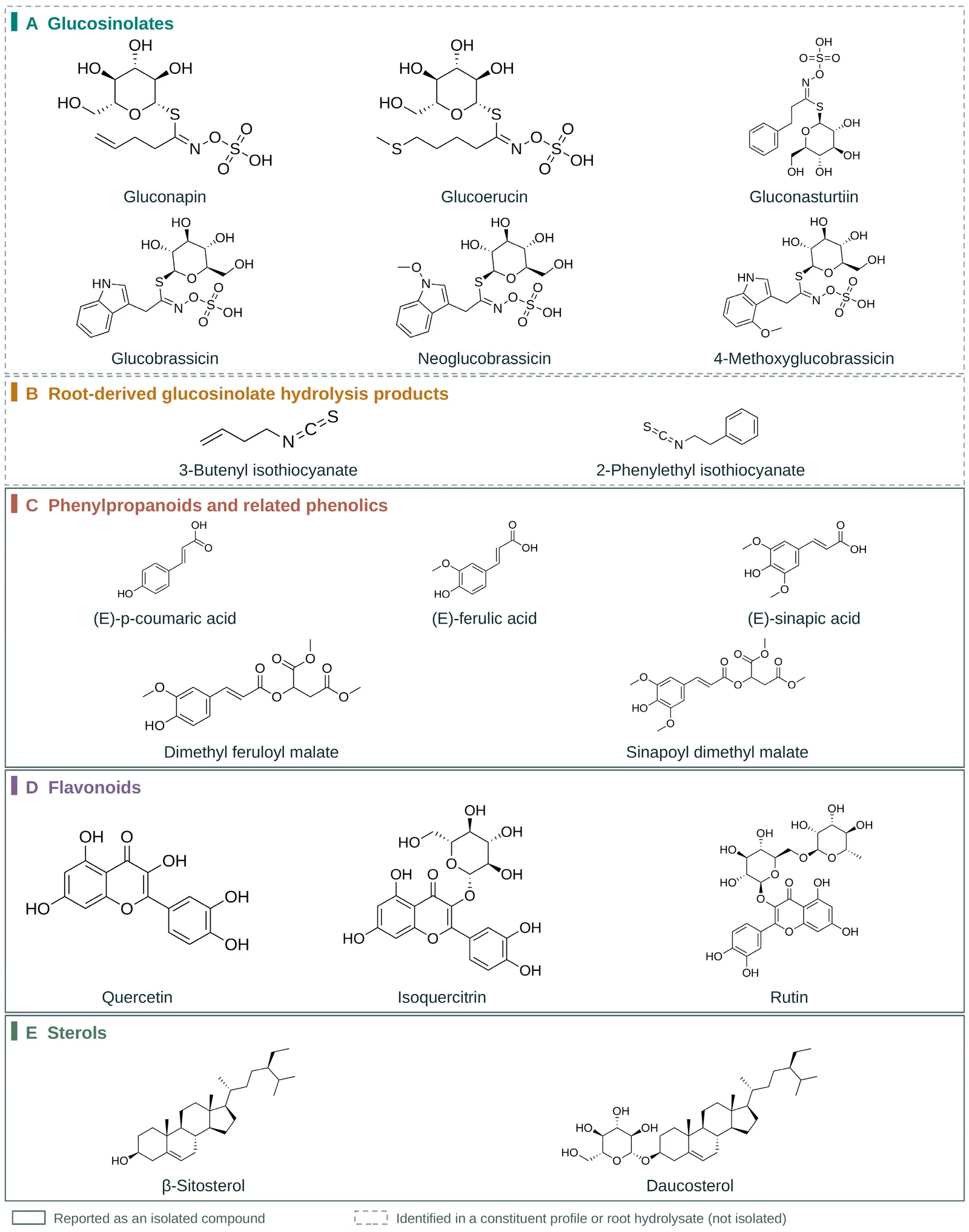
Chemical structures of representative small-molecule constituents reported in Qiamagu materials and glucosinolate hydrolysis products detected in Qiamagu root hydrolysates. Solid borders indicate isolated compounds; dashed borders indicate compounds identified in constituent profiles or root hydrolysates but not isolated from the corresponding material.

Composition studies reported glucosinolates and their hydrolysis products, phenolic acids and flavonoids, polysaccharides, lipids and sterols, amino acids, minerals, and volatiles in fleshy roots, root peels, seeds, aerial tissues, and derived fractions (Table S2; Figure 4 and Figure 6). Profiles varied with plant part, accession, treatment, and preparation, indicating that both material and analytical method shape the current understanding of turnip composition.

Constituent evidence was reported at four analytical levels: structural identification of isolated compounds, targeted analysis, library-based annotation, and class-level measurement. Isolated compounds characterized by nuclear magnetic resonance (NMR) or high-resolution mass spectrometry (HRMS), together with analytes confirmed against authentic standards, provided the most specific evidence of compound identity. Library matching provided tentative annotations, whereas assays reported as standard equivalents quantified constituent classes rather than individual compounds [43,44,61,62].

#### 3.3.1. Glucosinolates and Hydrolysis Products

Studies distinguished intact glucosinolate profiles, class-level totals, and hydrolysis products. In a common-garden comparison of 59 *Brassica rapa* accessions, PdCl_2_ colorimetry yielded total glucosinolates of 0.106–1.29 mg/g dry weight (DW), expressed as sinigrin equivalents, while liquid chromatography–mass spectrometry (LC–MS) annotated 10 glucosinolates and identified gluconasturtiin as a prominent profile feature [63]. The colorimetric result represents a class-level total, whereas the LC–MS result is primarily a semiquantitative profile. Because the accessions were grown together, the design supports differences among accessions under common cultivation rather than effects of climate or elevation at their source locations. Method dependence was also evident when three hydrolysis–extraction procedures applied to the same seed material yielded 2-phenylethyl isothiocyanate values of 11.93, 81.60, and 116.37 µg/g [61]. Glucosinolate measurements should therefore be compared only when the analyte and the hydrolysis, extraction, and analytical procedures are comparable.

#### 3.3.2. Phenylpropanoids, Phenolic Acids, and Flavonoids

Three forms of evidence were reported for phenylpropanoids, phenolic acids, and flavonoids: structural identification, relative metabolomic profiling, and class-equivalent assays. A study of fresh fleshy roots from Keping documented collection and botanical authentication, deposited a voucher specimen, and isolated 25 compounds whose structural assignments were supported by NMR and HRMS, including *p*-coumaric, ferulic, and sinapic acids and their derivatives [43]. These findings identify constituents in that batch but not their frequency or abundance in other materials.

A study of green- and purple-skinned cultivars compared anthocyanin-related signals across development, with cultivar and peel color varying together; the results therefore apply to the corresponding cultivar–color combinations [16]. Other studies measured total phenolics or total flavonoids using gallic acid, rutin, or other calibration standards [44,64]. Because these measures are based on different analytical principles, their numerical values are not directly comparable across studies.

#### 3.3.3. Polysaccharides: Preparation Level and Structural Evidence

The term “turnip polysaccharides” was applied to water-extracted and alcohol-precipitated crude preparations, chromatographic fractions, and purified polysaccharides. Hot-water extraction, microwave–ultrasound treatment, purification, and analytical procedures affected extraction yield and measured properties, while crude preparations could also contain proteins, phenolics, or low-molecular-weight coextractives [65,66,67]. Extraction yield therefore describes recovery under specified extraction and purification conditions; bioactivity comparisons require preparations with comparable composition and purification.

Detailed structural evidence was available for only a few purified fractions. Linkage and spectroscopic analyses of two neutral polysaccharide fractions, BRNP1 and BRNP2, supported locally branched α-(1→4)-linked D-glucopyranosyl (Glcp) motifs involving O-6 substitution [68]. A separate fraction, BRP-1-1, had a different monosaccharide composition and proposed linkage pattern, although its source reports differed in linkage proportions, spectral annotation, and the identity of the material tested in animals [66,69]. A galacturonic-acid-rich fraction supported an α-(1→4)-linked D-galactopyranuronic-acid (GalpA) main-chain segment [70]. These data support partial structural assignments of individual fractions rather than a shared complete structure of “turnip polysaccharide.” Direct comparisons among compositionally matched fraction series are still needed to evaluate structure–activity relationships (Table S2 and Table S4).

#### 3.3.4. Other Specialized Metabolites and Seed Lipids

Studies that isolated constituents from specific root and seed batches identified β-sitosterol, daucosterol, selected triterpenoids, and long-chain compounds. Several earlier structural assignments were supported by limited spectroscopic data [71,72]. One proposed seed-glycoside assignment could not be confirmed from the reported spectroscopic data [73].

Several seed-oil studies focused on erucic acid, with estimates differing by analytical method [74,75]. For erucic acid in turnip seed oil, gas chromatography–mass spectrometry (GC–MS) gave relative proportions of 48.21% and 47.67% in petroleum ether and diethyl ether extracts, respectively, whereas precolumn-derivatization high-performance liquid chromatography (HPLC) reported a content of 28.2% [74]. These values characterize specific batches and methods. Evaluation for food use also requires the complete product composition and the food-regulatory limits applicable in the intended jurisdiction.

#### 3.3.5. Nutritional Composition, Dietary Fiber, and Minerals

Fresh-root studies measured moisture, protein, sugars, vitamin C, amino acids, and major minerals [76,77]. A common-garden study compared nutritional traits across accessions and plant tissues [78]. Field sampling and comparisons among source regions also showed compositional differences among the materials examined, although genetic and environmental influences varied together in these designs [79,80,81,82]. The available common-garden comparison was conducted at a single site in one season; multi-site or multi-season common-environment studies would better distinguish accession and environmental contributions. Two dietary-fiber studies used enzyme–alkali or acid–alkali isolation, so their yields and swelling or water-holding properties describe the recovered fractions rather than total dietary fiber in fresh turnip [83,84].

Some nutritional measurements applied to seeds or experimentally treated plants rather than untreated fresh roots. Twelve free amino acids in mature seeds totaled 0.54% of dry weight; this value describes free amino acids in seeds, not the total amino acid composition of seed protein or the fleshy root [85]. Pot experiments with selenate or selenite altered selenium concentrations in roots and leaves under specified biofortification conditions [86]. Estimating nutritional exposure from untreated fresh roots will require representative samples analyzed with methods appropriate to that matrix.

Food-safety evaluation also requires measurements of contaminants, including heavy metals, and assessment of dietary exposure. These aspects were not evaluated in this review.

#### 3.3.6. Volatiles and Cross-Study Comparability

Volatile profiles were particularly sensitive to sample preparation and analytical platform. GC–MS and gas chromatography–ion mobility spectrometry (GC–IMS) detected 96 and 94 features, respectively, in the same purple, yellow, and white freeze-dried turnip slices, with only 15 features shared between the two platforms [87]. The results indicate complementary rather than equivalent analytical coverage. Compound identification is strengthened by authentic standards and retention indices, while odor-activity values and appropriately designed sensory tests are needed to establish aroma contribution. Analyses of roots, seeds, flowers, and different cultivars further showed that plant part, pretreatment, and platform are central to the interpretation of volatile profiles (Table S2).

Taken together, the evidence identifies glucosinolates and their hydrolysis products, phenylpropanoids, flavonoids, polysaccharide fractions, seed lipids, nutrients, and volatiles as the principal constituent groups studied in turnip (Table 1). Reported profiles varied with accession, tissue, developmental stage, processing, and analytical method. Within-study comparisons provide the clearest evidence for these sources of variation. Quantitative cross-study comparisons are often limited by differences in analytes, sample preparation, calibration, units, and measurement bases. For example, several total-phenolic and vitamin C results could not be reconciled by unit conversion [44,88].

### 3.4. Processing, Product Development, and Quality Changes

Processing studies fell into two related groups. Extraction and fractionation studies optimized the recovery of polysaccharides, phenolics, glucosinolates, saponins, seed oils, or fiber and primarily addressed ingredient preparation. Food-processing studies examined changes in edible matrices during cooking, drying, fermentation, pickling, formulation, preservation, and storage [65,66,75,83]. Extraction studies therefore focused on recovery and ingredient composition, whereas food-processing studies assessed composition, sensory quality, texture, microbiological quality, and storage stability in edible products.

**Figure 7:**
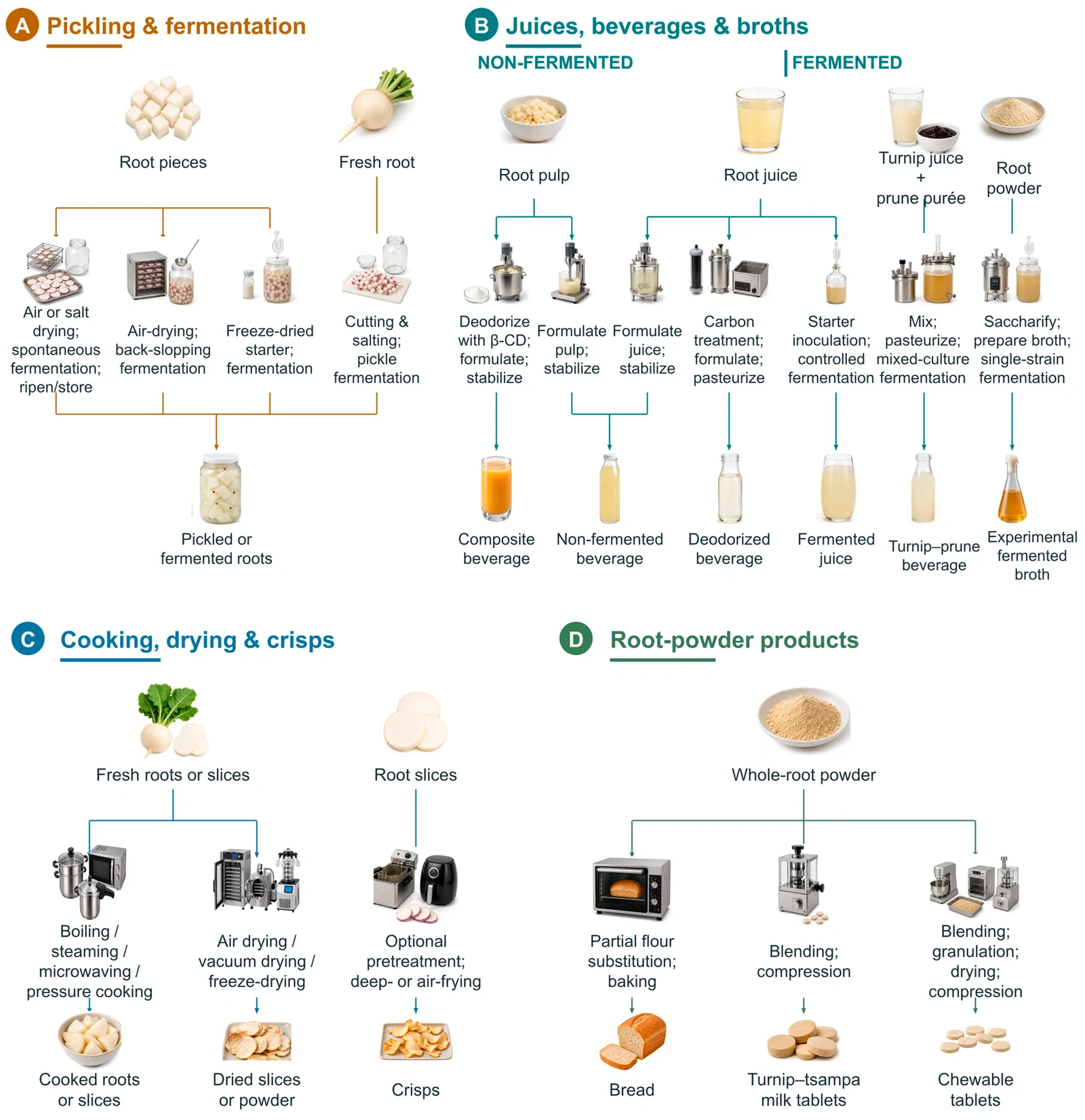
Selected processing routes and product forms reported for turnip. Branches represent alternative routes described in different sources. Selected illustrative elements were generated using OpenAI image-generation tools. β-CD, β-cyclodextrin.

Twenty-six reports, corresponding to 23 studies, directly evaluated food processing or product development. These studies covered fermentation or pickling, thermal processing, drying, product formulation, and storage (Table S3), and Figure 7 illustrates selected processing routes. Across these studies, quality changes depended on product form, processing conditions, and the attributes measured. Regional-use sources provided practical context for the foods and processes examined.

#### 3.4.1. Fermentation and Pickling

Spontaneous or regionally documented fermentation studies followed changes within specific recipes, whereas controlled studies compared defined starter cultures. Across both types, acidity, texture, microbial composition, volatiles, sensory attributes, and chemical composition changed along different trajectories. In one comparison, air-drying and salt dehydration produced different textures and compositions, although the independence of the fermentation batches was not clearly reported [39]. A 20-day low-salt fermentation study documented time-dependent changes in acidity, texture, volatiles, and microbial communities [9]. In a separate 28-day pickling study, the total sensory score peaked on day 14, whereas the relative ester peak area peaked on day 21 (40.56%) [46]. Thus, the preferred fermentation time depends on the quality attribute selected for the finished product.

Two controlled-fermentation studies compared four strains each. In one study, yeast J4-1 yielded comparatively favorable flavor scores, 2,2-diphenyl-1-picrylhydrazyl (DPPH) and hydroxyl-radical-scavenging results, and total antioxidant capacity, whereas other strains produced greater superoxide-anion-scavenging activity. The second study selected a strain identified in the original report as *Lactobacillus rhamnosus* CICC6154 on the basis of acidification, phenolic, volatile, sensory, and static-digestion recovery measures [92,93]. Neither study included a time- and temperature-matched uninoculated control, so the results primarily distinguish the tested strains.

A metabolomic study included juice, time- and temperature-matched uninoculated, and inoculated groups, but used only three samples per group and provided limited support for compound identification [94]. Compared with fresh Qiamagu juice, a fermented formulation containing Qiamagu and prune purée showed lower relative signals for sulfur-containing volatiles and a higher sensory score, while total phenolics and total flavonoids did not increase significantly [95]. Because prune addition and fermentation status changed together, these findings apply to the complete formulation and process.

#### 3.4.2. Thermal Processing and Drying

Thermal processing affected different endpoints in different directions. One study compared 2 mm slices boiled at 100 °C for 20 min, microwaved at 900 W for 5 min, or steamed for 10 min. DPPH radical-scavenging activity decreased after all three treatments, whereas cupric reducing antioxidant capacity (CUPRAC) increased after microwaving and steaming but decreased after boiling. The study used three roots per group; heat load and water contact differed among treatments, and the boiling liquid was not included in the mass balance [35]. Under the tested conditions, the contrasting DPPH and CUPRAC responses indicate that the apparent effect of heating depended on both the process and the assay used.

Freeze-drying and hot-air drying markedly reduced the relative signal for 2-phenylethyl isothiocyanate, whereas ester signals changed in different directions [96]. A comparison of five drying procedures for Qiamagu powder characterized powder properties under the specified equipment and conditions, but fresh-material baselines and common measures of processing time or energy use were unavailable [38]. Other laboratory-scale studies characterized powder properties and drying performance under defined process conditions [38,97,98].

#### 3.4.3. Product Formulation and Quality Evaluation

Product-development studies primarily evaluated composite beverages and prototype foods containing turnip ingredients. Activated-carbon deodorization improved selected odor measures while reducing total sugar, total phenolics, and in vitro xanthine oxidase (XO) inhibition, whereas increasing turnip-powder substitution impaired selected bread quality attributes [99,100]. Studies of milk tablets and yak meat sauce reported the composition and sensory performance of the complete formulations [101,102]. Orthogonal-array and response-surface designs further optimized processing conditions within the original formulations and scoring systems.

Storage and shelf-life evidence was product specific. Some studies reported selected microbiological measures, while others relied on physicochemical indices, model estimates, or extrapolation from observations made between days 0 and 84 [39,95,103]. These findings describe quality changes under the corresponding product conditions. Commercial shelf-life decisions additionally require product-specific failure thresholds and microbiological validation across the intended storage period.

Across studies, fermentation time and starter culture, water contact and heat load, and drying route were the principal process variables. Compositional, sensory, textural, microbiological, and storage endpoints did not always change in parallel. Only one study directly evaluated static digestion of a fermented turnip food; the remaining digestion-related literature concerned isolated fractions, delivery systems, or ex vivo fermentation [93] (Table S3 and Table S4). Overall, processing studies characterize product-quality changes more clearly than bioaccessibility after digestion.

### 3.5. Biological Activities

Most biological studies tested extracts, fractions, or multicomponent formulations rather than turnip foods, using chemical and enzyme assays, cell and ex vivo experiments, or animal models (Table S4). The findings below are grouped by biological response, with the tested preparation identified to distinguish activity of the tested preparations from effects of food consumption.

#### 3.5.1. Hypoxia and Exercise-Related Responses

Animal studies tested polysaccharide fractions, glucosinolate-loaded particles, and extracts in swimming-endurance and acute-hypoxia models. Medium and high doses of the incompletely characterized BRP-80 polysaccharide fraction increased swimming time to exhaustion [104]. Glucosinolate-loaded particles also increased swimming time at low and high doses, although a free-glucosinolate comparator was absent [105]. Acute-hypoxia responses varied in direction across doses and studies [106].

An aqueous-extract study reported metabolic and mitochondrial signaling changes but did not assess endurance [107]. A hypoxia-inflammation study reported hemodynamic and tissue changes alongside calcium-signaling associations [108]. The clearest functional signal was longer swimming endurance with selected preparations; acute-hypoxia responses varied.

#### 3.5.2. Neural Injury and Neurobehavior

Neural studies primarily tested pretreatment in ischemic or oxidative injury, sleep deprivation, and chemically induced cognitive impairment [109,110,111,112].

Linked animal and cell studies examined AET, a residual aqueous fraction obtained by partitioning combined ethanol and water extracts of authenticated roots. Four-week AET pretreatment improved selected neurological outcomes and reduced brain atrophy volume by approximately 51% relative to vehicle [33]. In linked HT22 oxygen-glucose deprivation/reoxygenation (OGD/R) experiments, LY294002 attenuated AET-associated improvements in viability and lactate dehydrogenase release, supporting involvement of phosphoinositide 3-kinase/protein kinase B/mechanistic target of rapamycin (PI3K/Akt/mTOR) signaling [113]. For the further-separated fraction AET-1, inhibitor testing assessed Akt/mTOR phosphorylation rather than functional rescue [114].

Other cognitive-impairment studies reported behavioral and tissue responses, with mechanistic hypotheses derived mainly from small cell experiments, metabolomics, or network analyses [109,111,112]. These findings chiefly concern pretreatment, particularly AET, rather than treatment after neural injury.

#### 3.5.3. Immune, Inflammatory, Respiratory, and Antimicrobial Responses

Polysaccharide preparations were tested in immunosuppression, acute lung injury, and macrophage-activation models [115,116,117]. Immune and intestinal responses varied by dose and accompanied changes in inflammatory-pathway gene expression and the microbiota [118]. Two purified neutral polysaccharides altered nitric oxide, tumor necrosis factor-α (TNF-α), and interleukin-6 (IL-6) release in RAW264.7 cells at relatively high concentrations [68]. An acidic polysaccharide was associated with inflammatory and redox changes in an acute lung-injury model [119].

Aqueous and *n*-butanol extracts altered cough, expectoration, asthma-related, or isolated-trachea responses, although extract standardization was incomplete [120,121]. Crude seed volatile oil inhibited tested bacterial strains, but inhibitory and bactericidal thresholds were reported as dilution factors rather than mass concentrations [122].

#### 3.5.4. Gut Microbiota

Studies of turnip polysaccharides linked microbial changes with barrier, inflammatory, or metabolic responses. One cyclophosphamide study used pooled fecal samples, so sequencing replicates did not represent independent animals [123]. In ex vivo fermentation with microbiota from eight donors, the relative abundance of *Bifidobacterium* increased from baseline with turnip polysaccharides but remained below the time-matched control [124].

Fecal microbiota transfer linked donor treatment with host responses under hypoxia, although microbial mediation remained unresolved [125,126]. A high-fat-diet rat study likewise linked microbiota and short-chain fatty acids with intestinal and lipid responses [127].

#### 3.5.5. Glucose, Lipid, and Urate Metabolism

Polysaccharide fractions and extracts altered glycemic, lipid, intestinal, and tissue-injury measures in high-fat-diet, streptozotocin, or combined metabolic-injury models [127,128,129,130]. Responses varied by preparation and dose, with small samples in several studies.

A 70% ethanol extract of turnip powder inhibited xanthine oxidase (XO) in vitro and altered organic anion transporter 1 expression in zebrafish; urate was not measured in that model [131]. Another crude extract was described as “Qiamagu saponins,” without identification of individual saponins. Reported decreases in both serum and urinary urate did not establish increased urate excretion, and dose and replication information was inconsistent [40,132].

#### 3.5.6. Tumor Models

In mice bearing Lewis lung carcinoma (LLC), a polysaccharide preparation reduced tumor weight by 22.3–40.3%, most strongly at the lowest dose. Direct exposure did not significantly reduce LLC or 4T1 mammary carcinoma cell viability, whereas macrophage coculture reduced tumor-cell growth, suggesting a host–cell contribution. Inhibitor experiments assessed macrophage markers rather than tumor-cell growth [133]. Crude polysaccharide plus cisplatin reduced H22 tumor growth, but the missing matched low-dose cisplatin-only control precluded a claim of synergy [134].

A petroleum-ether subfraction reduced A549 viability; antioxidant and caspase-inhibitor treatment partly reversed this response [135]. In esophageal-cancer cells, changes under pharmacologic modulation in apoptosis, viability, and migration were inconsistent [136]. These findings suggest differences between direct cytotoxicity and host-associated responses among preparations.

#### 3.5.7. Oxidative Stress, Aging, and Organ Injury

Oxidative-stress studies tested extracts and fractions in chemical assays, injured cells and tissues, and survival models. Colored-root-peel comparisons combined chemical and cell assays, with inconsistent reporting of total polyphenols [44].

Lifespan findings came from two possibly overlapping fruit-fly reports and one nematode study, with incomplete material and survival-analysis reporting [137,138,139]. D-galactose mouse studies assessed short-term injury and oxidative-stress outcomes rather than natural lifespan [140,141].

In hepatic-injury models, extract fractions and crude polysaccharides altered aminotransferase and tissue-injury measures, although preparations and doses differed [142,143]. Ethanol-injured HepG2 cells showed functional and PI3K/Akt-related changes, but inhibitor methods and controls were insufficiently reported [144,145,146]. In doxorubicin-injured H9c2 cells, ML385 partly attenuated polysaccharide-associated protection, supporting involvement of nuclear factor erythroid 2-related factor 2 (Nrf2) [147].

#### 3.5.8. Cross-Model Mechanistic Synthesis

The strongest mechanistic evidence concerns cell survival. Pharmacologic inhibition implicated PI3K/Akt/mTOR signaling in AET-associated protection of injured HT22 cells [113]. Nrf2 inhibition also weakened polysaccharide-associated protection of injured H9c2 cells [147]. Conversely, antioxidant and caspase-inhibitor treatment partly reversed petroleum-ether-subfraction cytotoxicity in A549 cells [135].

Across other preparations, pathway changes were chiefly associative, and functional tests of proposed mechanisms were incomplete or inconsistent [114,125,126,133,136,144,145,146]. Redox and inflammatory changes accompanied tissue protection, whereas metabolic and microbiota changes occurred alongside broader host responses. The contrasting cytoprotective and cytotoxic findings above also indicate that biological responses depend on the preparation and cellular context. Whether comparable responses occur after consumption of turnip foods remains unresolved (Table 2 and Table S4).

### 3.6. Human Evidence

After linked reports were grouped by participant population, the 12 records reporting human outcomes corresponded to eight participant populations (Figure 3B). Most studies concerned hypoxia or exercise or supportive care during chemotherapy, with substantial variation in participants, preparations, comparators, and observation periods. An uncontrolled mumps case series also reported human outcomes, but turnip was one of approximately 16 herbal ingredients and the results apply to the complete formulation [149].

#### 3.6.1. Hypoxia and Exercise

Human evidence on hypoxia and exercise comprised a short active-comparator trial, two 21-day studies, a small before-after comparison, and a single-case observation. A 7-day study reported as randomized and single-blind compared turnip powder with radish powder. It described within-group, sex-stratified changes in oxygen saturation, time to anaerobic threshold, and redox measures, but found no significant between-group difference in time to the target heart rate. Random-sequence generation and allocation concealment were not reported, and group allocation was unbalanced (18 versus 9) [150,151].

Two small five-arm studies included a *Rhodiola* active comparator and a starch placebo. A linked report for one study presented only three capsule arms, whereas the other evaluated fresh turnip, freeze-dried crisps, and a turnip/bamboo-leaf beverage [152,153,154]. Neither study showed a significant improvement in simulated-hypoxia tolerance time, although some superoxide dismutase, catalase, or malondialdehyde measures changed. Blinding was absent or incompletely reported, attrition occurred in some groups, and products containing bamboo-leaf flavonoids did not permit turnip-specific attribution. A before-after study of eight athletes found no significant change from baseline for either the turnip powder or the multicomponent beverage [155]. In a single high-altitude observation, minimum oxygen saturation increased slightly, whereas the apnea-hypopnea index increased and sleep efficiency decreased [156]. These studies document short-term physiological and redox changes, but no hypoxia-tolerance or exercise-performance effect has been replicated independently.

#### 3.6.2. Supportive Care During Chemotherapy

One study conducted during folinic acid, fluorouracil, and irinotecan (FOLFIRI) chemotherapy was reported as randomized, double-blind, double-dummy, and placebo-controlled; 64 participants were randomized and 61 were analyzed. A linked preliminary report described 30 participants from the same study series. Karnofsky Performance Status and some T-cell subset measures differed between groups, whereas interleukin-15 did not [157,158].

Another study allocated participants according to odd or even admission numbers and did not use a placebo. Between-group differences were reported for some symptoms, Karnofsky Performance Status, or immune measures, whereas several other outcomes did not differ significantly and white blood cell counts decreased in both groups [159,160]. Both studies assessed short-term supportive-care outcomes during specific chemotherapy regimens rather than tumor control.

Across the human studies synthesized here, some short-term physiological, redox, functional-status, and immune outcomes changed. However, no functional benefit has yet been replicated across human studies of a clearly defined turnip food or ethnomedicinal preparation (Table 3).

### 3.7. Future Research Directions

An immediate priority is to document current regional uses and verify the identity of the corresponding plant materials and preparations. Existing sources describe Uyghur foods and dietary therapy in Xinjiang, Yi fermented yuangen food in Liangshan, and fermentation, pickling, drying, or cooking in several other settings [3,6,9,39,47,49]. Contemporary surveys can determine whether these practices persist and document users, plant parts, preparation steps, and intake patterns. The current preparation and use of Tibetan medicinal turnip paste also warrant verification, together with assessment of compositional and stability variation among batches [10,11]. Beverages, baked foods, and other new formulations that differ materially from regional preparations should be evaluated as distinct finished products.

Processing research should determine how practically relevant processing conditions alter finished-food quality. Head-to-head comparisons using matched raw materials should relate compositional changes to sensory quality, texture, microbiological quality, and storage stability. Products showing reproducible process-dependent changes can then be examined for constituent release and bioaccessibility during digestion.

For health research, the central priority is to reproduce functional effects using preparations with traceable provenance, defined composition and dose, and models and primary outcomes aligned with the intended use. The human studies synthesized here identify two settings for better-controlled follow-up: hypoxia or exercise and supportive care during chemotherapy (Table 3). If pursued, hypoxia or exercise studies should test products prepared from the fleshy root without other active ingredients and use hypoxia tolerance or exercise performance as primary outcomes. Supportive-care studies should keep Qiamagu capsules used during FOLFIRI distinct from turnip-honey paste used during FOLFOX4 (leucovorin, fluorouracil, and oxaliplatin) or other regimens and prespecify clinically relevant outcomes such as symptom burden, functional status, treatment completion, and adverse events.

### 3.8. Strengths and Limitations

This review combines regional records of food and ethnomedicinal use with an in-depth synthesis focused on Chinese regional turnip materials and distinguishes edible tissues and finished foods from extracts, purified fractions, and multicomponent formulations. This material-level distinction permits compositional and processing findings to be interpreted for the corresponding food or product and health-related findings to be attributed to the preparation and model examined. The evidence is most coherent for turnip composition and for changes in finished products produced by fermentation, pickling, thermal processing, and drying.

Regional sources covered parts of Europe, Asia, North Africa, and the Caucasus, but they often provided incomplete information on contemporary users, frequency and amount of use, and variation in preparation. The detailed findings primarily concern the regional food and ethnomedicinal materials examined and do not provide a cross-country comparison of composition, processing, or health outcomes. Coverage of other-language literature also depends on database indexing and the availability of searchable English titles or abstracts. Botanical verification was also incomplete for some materials identified from regional names, plant parts, uses, and provenance. The review therefore cannot determine the prevalence or representativeness of individual practices; contemporary community and regional food surveys are needed to address these questions.

Experimental comparability was limited by variation in material identity, preparation characterization, models, comparators, and outcomes. Only a small number of cell studies reassessed functional outcomes after perturbing a candidate mediator; most mechanistic interpretations relied on pathway markers, omics, or associative findings. Human evidence was limited, methodologically heterogeneous, and concentrated on hypoxia or exercise and supportive care during chemotherapy. Results from concentrated extracts, isolated fractions, or multicomponent formulations cannot be attributed directly to ordinary turnip foods. Human evaluation requires defined foods or preparations and replicated functional outcomes.

## 4. Conclusions

The international literature documents turnip as both a food and an ethnomedicinal resource. For the regional materials examined in detail, the clearest findings concern material-specific composition and process-dependent changes in food quality. Fermentation, pickling, heating, and drying alter constituent profiles and sensory or storage properties. Extracts, fractions, and multicomponent formulations have shown biological activity in experimental models, but these findings do not establish health benefits from consuming turnip foods.

Human studies have not yet established a reproducible benefit for a defined turnip food or preparation. Linking documented uses with characterized raw materials and finished products offers a practical basis for further processing and health research. This review supports the development and evaluation of turnip foods while distinguishing established compositional findings from health effects that require further testing.

## Figures and Tables

**Table 1:** Compositional and nutritional evidence for turnip materials.

Constituent Class	Materials and Analyses	Representative Findings	Interpretive Considerations	References
Polysaccharides	• Material: fleshy-root extracts, fractions, and purified polysaccharides • Analysis: composition, glycosidic linkage, and spectroscopy	BRNP1 (~6.9 kDa) and BRNP2 (~4.8 kDa): locally branched α-(1→4)-Glcp with O-6 substitution BRP-1-1 (~31.4 kDa): Man:Glc = 7.62:1 Acidic fraction: α-(1→4)-GalpA segment	Preparation-specific structures Structure–activity relationships not directly tested	[66,68,70]
Glucosinolates and isothiocyanates/hydrolysis products	• Material: common-garden accessions and seed hydrolysates/extracts • Analysis: intact glucosinolates and hydrolysis products	10 glucosinolates across 59 accessions; total glucosinolates, 0.106–1.29 mg/g DW (sinigrin equivalents) Apparent 2-phenylethyl isothiocyanate, 11.93–116.37 µg/g across three seed procedures	Assay- and procedure-dependent values Not equivalent to native-tissue concentrations	[61,63,89]
Flavonoids, phenolic acids, and sinapoyl derivatives	• Material: voucher-backed roots and green/purple peels • Analysis: isolation, relative profiling, and class-equivalent assays	25 compounds isolated from one Keping batch Anthocyanin-related signals varied by cultivar and developmental stage	Single-batch compound identification Relative signals and class-equivalent values are not absolute concentrations	[16,43,64]
Other specialized metabolites and lipids	• Material: voucher-backed root isolates and mature-seed oil • Analysis: compound isolation and seed-oil fatty-acid profiling	Root sterols and long-chain lipids 92.4% unsaturation among six targeted seed-oil fatty acids	Profiles apply to the corresponding tissues and batches	[43,90]
Volatile and aroma-related chemistry	• Material: matched purple, yellow, and white freeze-dried slices • Analysis: GC–MS and GC–IMS	GC–MS and GC–IMS detected 96 and 94 features, respectively, with 15 shared.	Platform-specific feature counts, not concentrations Aroma contribution unconfirmed	[87]
Nutritional constituents and minerals	• Material: root powder, seeds, field tissues, and selenium-treated pot material • Analysis: proximate, amino-acid, mineral, and selenium measurements	Seed batch: 12 free amino acids, 0.54% DW Other studies: site- and plant-part variation; treatment-induced selenium accumulation	Single-batch/site evidence Heterogeneous materials and designs Selenium enrichment does not represent baseline content or dietary intake	[80,85,86,91]

Note. Values summarize representative within-study findings. Cross-study pooling was not undertaken because materials, methods, units, calibration, and reporting bases differed. Isolated compounds, library matches, relative signals, and class-equivalent assays represent distinct analytical outputs. BRNP1, BRNP2, and BRP-1-1 are identifiers used in the original reports. Abbreviations: DW, dry weight; GalpA, galactopyranuronic acid; GC–IMS, gas chromatography–ion mobility spectrometry; GC–MS, gas chromatography–mass spectrometry; Glc, glucose; Glcp, glucopyranosyl; kDa, kilodaltons; Man, mannose.

**Table 2:** Nonhuman evidence on biological activities and selected mechanistic findings of turnip preparations.

Evidence Area	Contributing Records, *n* *	Test Materials and Study Models	Representative Findings	Design and Interpretive Considerations	Representative References
Hypoxia and exercise-related responses	48	• Materials: extracts, polysaccharides, and glucosinolate-loaded particles • Models: hypoxia, exercise, and cellular stress	• Responses varied by dose and endpoint • High-dose glucosinolate-loaded particles increased swimming time (17.4 vs. 12.7 min) • A separate chronic study assessed metabolic and signaling measures without an endurance endpoint	• Heterogeneous preparations and models • No free-glucosinolate comparator	[105,106,107]
Neural injury and neurobehavior	21	• Materials: residual aqueous fraction AET and a further-separated fraction • Models: mouse cerebral ischemia–reperfusion and linked HT22 OGD/R	• Four-week AET pretreatment improved selected neurological outcomes • Brain-atrophy volume was ~51% lower than with vehicle • LY294002 attenuated AET-associated changes in HT22 viability and LDH release	• The further-separated fraction was reassessed for Akt/mTOR phosphorylation but not functional rescue	[33,113,114]
Immune responses	32	• Material: polysaccharide fractions • Models: immunosuppressed mice and macrophages	• Selected immune measures shifted partly toward control values in mice • At the highest dose, natural-killer-cell activity did not differ significantly from the model group • Macrophage cytokine changes occurred only at high concentrations	• Endotoxin and receptor/genetic controls were not reported	[68,118]
Gut microbiota and metabolism	33	• Material: polysaccharides • Models: animal studies, ex vivo human-fecal fermentation, and fecal microbiota transplantation	• Microbiota, short-chain fatty acids, barrier indices, and host measures changed concurrently • Ex vivo *Bifidobacterium* relative abundance remained below the time-matched control • Fecal-microbiota-transfer studies produced partial or nonsignificant host responses	• Sample pooling and incomplete mediation controls limit causal attribution	[123,124,125,127]
Tumor models	17	• Materials: polysaccharides and crude-polysaccharide/cisplatin combinations • Models: tumor-bearing mice, cancer cells, and macrophages	• Tumor weight was 22.3–40.3% lower than with vehicle, with the largest reduction at the lowest dose • No significant decrease in LLC or 4T1 cell viability	• The cisplatin comparison did not distinguish additivity from synergy • Mediator testing was incomplete	[133,134]
Glucose, lipid, and urate metabolism	22	• Materials: polysaccharides and aqueous/ethanol extracts • Models/assays: glycemic and lipid models, XO assays, and zebrafish/mouse hyperuricemia	• Selected glucose and lipid endpoints changed • XO inhibition was reported • Urate and OAT1 findings varied by model and included inconsistencies in the source reports	• Small samples • Incompletely defined crude extracts • Dose and unit inconsistencies	[40,127,128,130,131,132]
Oxidative stress and aging	67	• Materials: crude extracts and candidate compounds • Models: chemical assays; cell and tissue systems; animal injury/aging models; *Caenorhabditis elegans*	• Tested root extracts altered selected lifespan, motor, stress-resistance, and redox endpoints • Responses differed across assay and model levels	• Mechanistic evidence was mainly associative • Preparations were not chemically normalized • Mediator testing was limited	[44,138,139,140,141,147,148]
Liver injury models	11	• Material: ethanol-extract fractions • Models: carbon tetrachloride-induced liver injury in mice and chemical assays	• Aminotransferase and redox-enzyme responses varied among fractions and doses • Histological findings were reported qualitatively	• Incomplete fraction characterization • Qualitative histology • High doses	[142]
Inflammatory responses and antimicrobial activity	25	• Materials: acidic polysaccharide and crude seed volatile oils • Models: lung injury and assays against three bacterial strains	• High-dose polysaccharide changed bronchoalveolar-lavage inflammatory and redox markers • Inhibitory and bactericidal thresholds for crude seed volatile oils were reported as dilution factors	• Incomplete endotoxin, mass-concentration, and component-attribution controls	[119,122]

Note. * Number of eligible bibliographic records contributing to each nonexclusive category (Table S4); counts describe evidence coverage, not independent replication or evidence strength. Source- and experiment-level details are provided in Table S4. Abbreviations: 4T1, murine mammary carcinoma cell line; AET, residual aqueous fraction obtained from combined ethanol and water extracts; Akt, protein kinase B; HT22, mouse hippocampal neuronal cell line; LDH, lactate dehydrogenase; LLC, Lewis lung carcinoma; mTOR, mechanistic target of rapamycin; OAT1, organic anion transporter 1; OGD/R, oxygen–glucose deprivation/reoxygenation; PI3K, phosphoinositide 3-kinase; XO, xanthine oxidase.

**Table 3:** Human studies of turnip materials and turnip-containing formulations.

Participants and Setting (n)	Design and Comparator	Turnip Material and Regimen	Main Findings	Design and Reporting Considerations	References
Healthy students *n* = 27 (turnip, 18; radish, 9)	Reported randomized, single-blind • Active comparator: radish powder	• Freeze-dried whole-turnip powder • 15 g/day for 7 days	• Within-group changes in oxygen saturation, time to anaerobic threshold, and redox measures • No between-group difference in time to target heart rate	• Unequal groups (*n* = 18 vs. *n* = 9) • Predominantly within-group analyses • Active comparator; no inert comparator	[150,151]
Healthy men aged 20–30 years 40 enrolled; 39 analyzed	• Baseline-stratified, randomized, five-arm • Starch placebo; *Rhodiola* active comparator • Blinding unclear	• Combination capsule, fresh turnip, or turnip/bamboo-leaf beverage • 21 days	• Changes in some SOD, CAT, and MDA measures • No between-group improvement in simulated-hypoxia tolerance	• Small study arms • Pooled reporting • Randomization and blinding incompletely described	[152,153]
Healthy men aged 20–30 years 50 enrolled; 39 analyzed	• Baseline-stratified, randomized, open-label, five-arm • Potato-starch placebo; *Rhodiola* active comparator	• Fresh turnip, 100 g/day • Crisps, 17 g/day • Turnip/bamboo-leaf beverage, 350 mL/day • 21 days	• No between-group improvement in hypoxia tolerance • Changes in some oxidative-stress measures	• 11 participants not included in analysis • Small open-label arms	[154]
Athletes *n* = 8 (4/group)	• Uncontrolled before-after product groups	• Turnip powder, 15 g/day • Multicomponent beverage, 268 mL three times/day • 7 days	• No outcome changed significantly from baseline in either product group	• *n* = 4 per group • Uncontrolled before-after analysis • Multicomponent beverage; turnip-specific effects not separable	[155]
Moderate obstructive sleep apnea-hypopnea Single case (*n* = 1)	• Sequential uncontrolled single-case observation	• Turnip powder, 15 g twice daily • Approximately 1 week	• Minimum oxygen saturation slightly increased • Apnea–hypopnea index increased • Sleep efficiency decreased	• Single case • Sequential uncontrolled exposure • Acclimatization not controlled	[156]
Advanced colorectal cancer during FOLFIRI 64 randomized; 61 analyzed	• Randomized, double-blind, double-dummy, placebo-controlled	• Qiamagu capsules, 15 capsules three times/day + FOLFIRI • 14 days	• Between-group differences in KPS improvement and some T-cell indices • No between-group difference in IL-15	• Inconsistent participant flow across linked reports • 14-day assessment period	[157,158]
Intermediate/advanced colorectal cancer 80 enrolled; 76 analyzed	• Allocation by admission-number parity (quasi-random) • No placebo	• Turnip–honey paste + chemotherapy • FOLFOX4 for 14 days or other regimens for 21 days	• Between-group differences in some symptoms, KPS, or immune measures • No difference for several other outcomes • White blood cell counts decreased in both groups	• Open allocation by admission-number parity • Multicomponent paste • Regimens and durations differed	[159,160]
Mumps outpatients *n* = 122	• Uncontrolled oral multicomponent regimen + external treatment	• Turnip root in an approximately 16-herb oral regimen • External co-treatment	• Reported outcomes: 120 recoveries, 1 hospitalization, and 1 discontinuation	• No comparator • Multicomponent regimen with co-interventions • Outcome definitions incompletely specified	[149]

Note. The 12 records were grouped into eight participant populations. Three records concerned two 21-day studies; because no evidence indicated that the studies shared participants, they were counted as two populations. Abbreviations: CAT, catalase; FOLFIRI, leucovorin, fluorouracil, and irinotecan; FOLFOX4, leucovorin, fluorouracil, and oxaliplatin; IL-15, interleukin-15; KPS, Karnofsky Performance Status; MDA, malondialdehyde; SOD, superoxide dismutase.

## Data Availability

The data supporting this scoping review are contained within the article and its Supplementary Materials. The review protocol and record-level geographic data underlying Figure 2 are available through an anonymized view-only link to a private Open Science Framework (OSF) project: https://osf.io/xv4zu/overview?view_only=914b9c4f3e7a4c4e931042e56412be08 (accessed on 11 October 2026). No new experimental or individual-level participant data were generated.

## Supplementary Materials

The following supporting information can be downloaded at: https://www.mdpi.com/article/10.3390/foods15203648/s1, File S1: Preferred Reporting Items for Systematic reviews and Meta-Analyses extension for Scoping Reviews (PRISMA-ScR) Checklist; Table S1: Bibliographic and evidence-map characteristics of included records; Table S2: Characteristics of sources reporting compositional and nutritional data; Table S3: Characteristics and findings of processing and product-development studies; Table S4: Characteristics and reported outcomes of charted nonhuman bioactivity experiments.
